# Concerning mobile phone use and risk of acoustic neuroma

**DOI:** 10.1038/sj.bjc.6603238

**Published:** 2006-06-27

**Authors:** J D Boice, J K McLaughlin

**Affiliations:** 1 International Epidemiology Institute, Rockville, MD 20850, USA; 2 Department of Medicine and Vanderbilt-Ingram Cancer Center, Nashville, TN 37232, USA

**Sir**,

In a recent letter criticising one of several IARC Interphone studies investigating possible associations between mobile phone use and brain tumour risk, Hardell and Mild (2006) claim that the International Epidemiology Institute (IEI) ‘has been linked to Motorola’. No such link has ever existed. Senior investigators at IEI have conducted research on mobile phone use and cancer (Inskip *et al*, 1999; Inskip *et al*, 2001; Johansen *et al*, 2001, 2002; Christensen *et al*, 2005), but this research was not funded by Motorola or by any other mobile phone manufacturer. The erroneous claim by Hardell and Mild appears linked to our review of the epidemiologic literature on cellular telephones and brain cancer (Boice and McLaughlin, 2002), which was prepared at the behest of the Swedish government with no funding by the mobile phone industry. Our review, like those of other senior scientists (Rothman, 2000, 2001; AGNIR, 2003; SSI, 2003; Ahlbom *et al*, 2004), raised methodologic questions about the case–control studies on mobile and cordless phone use and brain tumour risk conducted by Hardell, Mild and colleagues. It is unfortunate that Hardell and Mild chose to respond to our evaluation of scientific issues with false innuendo.

**Editors Note** The Editor-in-Chief of *British Journal of Cancer* now considers the correspondence published in Volume 94 Issue 9 and above closed.
